# Epidemic Erysipelas

**Published:** 1845-02

**Authors:** 


### EPIDEMIC ERYSIPELAS.

We perceive by the public papers that this serious malady continues to visit different neighborhoods and often proves fatal. It appears to prevail more in winter than in summer, and to affect the country more than the towns, especially the larger. Louisville for the year past has given evidence of the existence within her limits of its remote cause, whatever that cause may be, but the number of cases has not been great. In the hospital, more have occurred than for several years before—some spontaneous, and some following surgical operations. Very lately, the extraction by Professor Gross, of a large tumor from the angle of the jaw of a negro man, from the country, in excellent health, was followed by severe, though not fatal erysipelas of the face and head. We hope our friends where the disease prevails will furnish us with accurate histories of it, for publication. Among the points to which we would especially direct their attention are, its connexion with puerperal fever, and its contagiousness D.

